# Congenital heart disease, pulmonary arterial hypertension and the UK’s Drivers and Vehicle Licensing Agency: controversial new guidance

**DOI:** 10.1177/2045894019882627

**Published:** 2019-10-30

**Authors:** Andrew Constantine, Robert Tulloh, Robin Condliffe, Paul Clift, Konstantinos Dimopoulos

**Affiliations:** 1 Adult Congenital Heart Centre and National Centre for Pulmonary Hypertension, Royal Brompton Hospital, London, UK; 2 National Heart and Lung Institute, Imperial College London, London, UK; 3 Bristol Heart Institute, University Hospitals Bristol NHS Foundation Trust, Bristol, UK; 4 Pulmonary Vascular Disease Unit, Royal Hallamshire Hospital, Sheffield, UK; 5 Department of Cardiology, Queen Elizabeth Hospital Birmingham, Birmingham, UK

**Keywords:** adult congenital heart disease, pulmonary hypertension, driving restrictions, UK guidelines

Recently, the UK’s Driver & Vehicle Licencing Agency (DVLA) published updated guidance for medical professionals on assessing fitness to drive.^1^ This included updates for adults with congenital heart disease (ACHD) and a new section for those with pulmonary hypertension. There is some overlap in these conditions, with pulmonary arterial hypertension (PAH) affecting 5–10% of congenital heart disease patients. Both PAH and ACHD are the remit of the specialist, and in both conditions social independence and psychological wellbeing are key goals of care. Driving can enhance an individual’s independence by facilitating engagement in social roles, including employment, family life and peer relationships. In the presence of medical conditions that can significantly affect exercise tolerance, this freedom must be balanced against the risk of a sudden disabling event.

In the new guidance, anyone with ACHD and symptoms, including any severity of palpitations or breathlessness, must stop driving immediately and must notify the DVLA. Patients with PAH who are under the care of a specialist centre may only drive after specialist assessment, and only if the assessment concludes that there is an annual risk of a disabling event of <20%. The requirements are even stricter for group 2 licence holders. Failure to disclose a medical condition that affects driving eligibility can lead to a fine of up to £1000, and criminal prosecution if driving results in a road traffic collision.

These changes are significant. Previously, patients with either condition could continue to hold a group 1 licence provided there was “no other disqualifying condition”. Under the new guidance, however, patients with symptomatic congenital heart disease may face a blanket ban on driving.

Inconsistencies in the guidelines disadvantage our patients. For example, palpitations are included in the list of prohibitive symptoms in ACHD. For non-ACHD patients with arrhythmia, driving must stop only if the arrhythmia has caused or is likely to cause incapacity. This creates a double standard – one rule for ACHD patients and one rule for everyone else.

For PAH, specialist assessment is required to ensure that the annual risk of a disabling event is <20%. However, current validated risk stratification models only assess overall mortality risk; none focus on sudden death or syncope.^2–6^ Trial outcomes in PAH do not report ‘annual disabling event rate’, which makes the provision of meaningful, evidence-based estimates a fool’s errand.

In addition to being evidence-based and fair, obligations placed upon the health service need to be feasible and cost-effective. Full implementation of this DVLA guidance, however, places an unrealistic burden on healthcare practitioners. Additional ‘specialist assessment’ of 5500 adults with PAH receiving follow-up in dedicated centres^7^ represents a large increase in workload. For ACHD specialists, who are faced with an estimated UK population of 120,000 patients,^8,9^ the task is even more colossal. Such ‘inclusive’ guidance requires clinicians to make an updated assessment of driving eligibility in every consultation, with little evidence on how to achieve this in an extremely heterogeneous population. In ACHD, the same symptom can have widely different implications depending on underlying anatomy. For example, palpitations due to atrial fibrillation in the context of a Fontan circulation signals a significantly increased one-year risk of death (non-sudden), whereas this is not the case for a patient with an atrial septal defect. Guidance has to be lesion-specific to reflect true risk. By the same token, symptoms that are not causing incapacity at the wheel should not affect one’s eligibility to drive unless there is a disease-specific cause for concern.

Ultimately, the limitation of the “law, marching with medicine but in the rear and limping a little” (Lord Justice Windeyer, 1970)^10^ should not be replaced by law racing ahead of scientific knowledge, leaving a trail of anxious patients and confused clinicians in its wake. The current guidance for ACHD and PAH is controversial and signals the immediate need for collaboration and consensus across experts nationwide. The goal should be to urgently put in place practical and easy-to-implement guidance, which reflects current medical knowledge and protects the public and the patient. Future studies are needed to inform DVLA guidance. In the meantime, we strongly recommend that current guidance is urgently revised seeking expert consensus opinion.
